# Human resources for health and decentralization policy in the Brazilian health system

**DOI:** 10.1186/1478-4491-9-12

**Published:** 2011-05-17

**Authors:** Celia Regina Pierantoni, Ana Claudia P Garcia

**Affiliations:** 1 Social Medicine Institute of Rio de Janeiro State University (IMS/UERJ), Rio de Janeiro, Brazil; 2 Human Resources for Health Observatory - Workstation of IMS/UERJ, Rio de Janeiro, Brazil; 3 Collabourating Center of the Pan-American Health Organization/World Health Organization (PAHO/WHO) for Health Workforce Planning and Information, Brazil

## Abstract

**Background:**

The Brazilian health reform process, following the establishment of the Unified Health System (SUS), has had a strong emphasis on decentralization, with a special focus on financing, management and inter-managerial agreements. Brazil is a federal country and the Ministry of Health (MoH), through the Secretary of Labour Management and Health Education, is responsible for establishing national policy guidelines for health labour management, and also for implementing strategies for the decentralization of management of labour and education in the federal states. This paper assesses whether the process of decentralizing human resources for health (HRH) management and organization to the level of the state and municipal health departments has involved investments in technical, political and financial resources at the national level.

**Methods:**

The research methods used comprise a survey of HRH managers of states and major municipalities (including capitals) and focus groups with these HRH managers - all by geographic region. The results were obtained by combining survey and focus group data, and also through triangulation with the results of previous research.

**Results:**

The results of this evaluation showed the evolution policy, previously restricted to the field of 'personnel administration', now expanded to a conceptual model for health labour management and education-- identifying progress, setbacks, critical issues and challenges for the consolidation of the decentralized model for HRH management. The results showed that 76.3% of the health departments have an HRH unit. It was observed that 63.2% have an HRH information system. However, in most health departments, the HRH unit uses only the payroll and administrative records as data sources. Concerning education in health, 67.6% of the HRH managers mentioned existing cooperation with educational and teaching institutions for training and/or specialization of health workers. Among them, specialization courses account for 61.4% and short courses for 56.1%.

**Conclusions:**

Due to decentralization, the HRH area has been restructured and policies beyond traditional administrative activities have been developed. However, twenty years on from the establishment of the SUS, there remains a low level of institutionalization in the HRH area, despite recent efforts of the MoH.

## Background

Brazil is a federal republic with 27 States and more than five thousand cities (if municipalities are included). Each state and their cities have political and administrative autonomy in the management of public policies. The National Health System consists of a funded public sector, the Unified Health System (SUS), and a private sector, comprising several prepayment mechanisms (e.g. health insurance) and out-of-pocket financing. The SUS is defined in the 1988 Brazilian Constitution as being founded on the principles of universal coverage, integral care and equity, with the aim of providing free access to health care for the whole population. It provides exclusive coverage for 78.8% of the Brazilian population. The remaining 21.2% of the population--covered by a supplementary system--also have the right to access services provided by SUS. Furthermore, the SUS is also responsible for the provision of services such as health surveillance, disease control and health industry regulation [1]. There are about 2 200 000 healthcare workers, most of them employed by the public sector, with many at the municipal level (Table 1).

**Table 1:** Health employment by administrative level, Brazil, 2002.

	Federal	Estate	Municipal	Total public sector	Total private sector
Number	96 064	306 042	791 377	1 193 483	987 115

%	4.4	14.0	36.3	54.7	45.3

Source: IBGE, Pesquisa Assistência Médico-Sanitária, 2002.

Although unified coordination is expected at every level of the administration (federal, state and municipal), in terms of responsibilities and prerogatives in the formulation of sectoral policies, the various contextual, organizational and economic conditions have influenced the modes of implementation and sustenance of the SUS.

The Brazilian health reform process put great emphasis on decentralization, transferring decision making authority to sub-national levels. Such decentralization requires political and administrative organizational structures to manage public policies with legally conferred, socially accepted, financial and administrative responsibilities.

Therefore, states and municipalities were given more responsibility in the development and implementation of human resources for health (HRH) policies following the reform, which allowed the formation of a 'new' arrangement of the health labour management and education area, at all levels. This new set-up can be defined as a set of activities that involve the planning, funding, recruitment, deployment, allocation and training of health workers. As a whole, this set of activities is aimed at improving health care quality and regulatory mechanisms (through cooperation between professional councils and associations, public and private health education institutions and civil society.

The reform brought policies to change the organization, operation and management of services, modifying working conditions and redefining roles and models of managing human resources (HR). In this context, the political and administrative decentralization process included a key component: providing greater freedom of choice between systems and services, which implied redefining and strengthening human resources management, especially in public services.

The consensus around these ideas emerged in the 1970s, with the exhaustion of the paradigm of the centralized public sector. In the HRH area, some functions which are decentralized are related to employment (hiring and firing, nature of tenure, defining the compensation package); to management (transfers, promotions and sanctions), and to skill-mix and training [2].

According to Fleury [3], the 1988 Brazilian Constitution broke new ground by building a democratic institution, focusing too much, however, on the public perspective, in contrast to the new global order, guided by globalization and neoliberalism. While other countries were already affected by the wave of neoliberal market logic as the guiding model of social reform, in Brazil the changes of the 1980s were marked by the decentralization of policies and services and the pursuit of a universal system of social protection, including health care.

The tendency to concentrate fiscal resources at the federal level was reversed with the political, administrative and financial decentralization. The mechanism for transferring federal funds for sub-national levels was also amended, and at the end of the 1980s more resources started to be transferred automatically, based on population and per capita income.

As pointed out by Melo [4], there is a strong polarization in the public debate in Brazil about this issue. Some consider the process as virtuous as, in addition to a more robust democracy, the strengthening of sub-national levels of government should improve allocating efficiency in the government system. Others, however, consider that states and municipalities are *loci *of patronage and inefficiency, so their empowerment results in ungovernableness. Furthermore, it is also argued that some efforts to stabilize the federal administration have brought fiscal irresponsibility to the lower levels.

It has been also noted that a positive factor of decentralization is that, theoretically, it involves the community in the promotion and management of services, allowing a simplification of procedures, facilitating the purchase of supplies and equipment, adaptation of services to local needs and improving HR administration, with greater accountability. In a suitable process of decentralization, there are changes at all levels of responsibility, reaching the smallest units and the most peripheral levels of decision making. However, the lack of institutional capacity at the local level and of clear instruments to coordinate and consolidate nationwide policies may compromise the advantages of decentralization [5].

Within this context of transition from a highly politically and economically centralized system to a decentralized system, the municipal administrations started to play a key role in the health arena. Noronha et all [6] point out that, as a way to achieve certain goals, decentralization was the only organizational guideline of the SUS that did not go against the so-called 'neo-liberal ideas' of strengthening the right to health and opposing the expansion of size of the state and

However, Pierantoni at al. [7], argue that the decentralization of health services in Brazil did not result automatically in the transfer of management capacity for municipal levels. In fact, it worsened chronic problems and, in accordance with the political demands, forced the municipal health managers to develop several different solutions and special administrative arrangements, including changes in the system, which generate constraints and legal challenges.

Vianna and Machado [8] show that the recent experience of forming a new political agreement at federal level revealed the importance of the federal administration in the formulation and regulation of public policies--something that it not incompatible with sectoral decentralization policies.

In Brazil, the implementation of the public health system was supported by two different approaches. The first one is the federal centralization that made a decentralization policy possible, in which the federal administration has the authority to define standards, financial incentives and other tools of national induction. This is possible because in the Brazilian health system there is a federal pact to cooperation among federal level, states and municipalities. The second approach was the support of social and political actors of highly organized sub-national authorities and federal managers.

As Dal Poz [9] has observed, in relation to the HRH policies, there was an almost automatic mirroring of what was established at the federal level by the other administrative levels; and a lack of innovation and adoption of policies that responded to specific problems. Even in municipalities with more innovative health policies, the behaviour of policy makers and health managers is considerably conservative. According to Dal Poz, in the late 1990s, there was a need to establish national policies that incentivized regional and local policy-makers and decision-makers to adopt policies better suited to their needs.

To address some of these challenges in the HRH area, in 2003 the Ministry of Health (MoH) created the National Secretary of Health Labour Management and Education (SGTES). Since then, the federal government has been formulating policies to guide health labour management, education, training and professional practice and regulation. This paper assesses whether the process of decentralizing human resources for health (HRH) management and organization to the level of the state and municipal health departments (SES and SMS) has involved investments in technical, political and financial resources originally allocated to health labour and education management at the national level (MoH).

## Methods

We combined quantitative and qualitative methods to better capture all dimensions of this issue. According to Minayo [10], there is no opposition between quantitative and qualitative data, but rather complementarity, reflecting the dynamic interaction within the reality they represent, eliminating any dichotomy.

Building on previous work [11,12], we conducted some workshops with researchers, master students, graduate trainees and HR consultants to develop a survey, with 74 questions, divided into five sections:

1. Characterization of health departments and managers.

2. Managers' level of knowledge about SGTES.

3. Health labour management policies.

4. Education management policies.

5. Managers' opinions regarding SGTES policies.

A pre-test was carried out in consultation with HRH management experts. Once the changes outlined and recommended in the pre-test were done, we disseminated the research to increase the managers' awareness and participation.

From the experience gained in previous research a computer-assisted telephone interview (ETAC) method was used. The data collection phase lasted five months, being completed in February 2008, with 253 HRH managers of 27 state health departments (SES) and 206 large cities (SMS, including 23 capitals).

After the ETAC, the database was cleaned and verified for consistency of the information collected. The responses were processed using specific software, *Sphinx *[13], allowing direct tabulation and statistical analysis of collected data.

The 'cut-off point' question was whether the creation of SGTES had generated or influenced changes in the departments surveyed (question 17). Thereafter, we analyzed the data obtained by comparing answers to the question of whether there were changes following the creation of SGTES with questions that examined the influence of federal level policy guidelines in HRH management in sub-national governments.

When analyzing results, we identified information deserving in-depth or further research, due to its importance to--or its relationship with--the our central research theme. Based on this, we decided to perform focal groups with HRH managers from five geographic regions of the country.

The focal groups were carried out from 6 March to 10 April 2008, conducted by two researchers. One was the moderator, explaining the purpose and format of the meeting so that participants knew what to expect. Another researcher played the role of rapporteur, recording the discussion through voice recording and taking notes regarding the content and behavior of participants. The information was systematized by region, then translated into a general framework noting the prevalent ideas or themes. This material was then further analyzed.

From the results obtained so far, a comparative study was carried out based on the results of the previous survey conducted by the National Council of State Health Departments (in 2004) [11], in the cities and capitals with more than 100 000 inhabitants [12]. We were then able to identify progress made, and setbacks and challenges encountered, indicating the trends in the SUS decentralization of HRH management.

## Results and discussion

From the broad set of health labour and education management policies assessed, we present in this paper the results of the analysis of the strategies considered most relevant to HRH decentralization, and to the structuring and organization of HRH management at state and municipal levels.

### Existence of an HRH unit

It was observed that 76.3% of the health departments have a human resources for health unit of some kind, as shown in Table 2. Most of them followed the federal model (SGTES), covering two areas: health labour and health education management.

**Table 2:** SMS/SES with HRH unit, Brazil, 2008.

HRH unit	number	%
yes	193	76.3

no	57	22.5

no response	3	1.2

TOTAL	253	100.0

Source: Pesquisa Gestão do Trabalho e da Educação em Saúde.

ObservaRH/IMS-UERJ. Brazil, 2008.

Almost half (48%) of the health departments have changed since 2003, following the establishment of the HRH unit (SGTES) at the Ministry of Health.

Organizational changes within the HR units at SMS and SES due to the policies implemented by SGTES were reported by 48% of the respondents. One of the major changes, mentioned by 62% of participants, was the participation in technical cooperation projects proposed by SGTES.

### Health labour management policies

Among the health labour management activities, the study looked at the career path proposed by the federal level, calling for a 'unified' career that would be similar at all levels. It was observed that 47.8% do not have any career path plans, particularly at municipal health departments outside the capitals; about 20% have a specific career plan for the health division and 29% have a plan for all civil service workers.

Another strategy analysed in the study was the Labour Negotiation Program, with about 27% of the managers involved. This program is an important tool for the SUS health labour management, ensuring participation of employees, employers, managers and administration representatives. It allows independent discussions on several aspects of the SUS labour relations and working conditions, such as hours of working, wage and career path.

The study also assessed the program for reducing the number of jobs with no labour rights or social protection (e.g. without social security, weekly paid rest period, vacations, etc.). It was observed that 42% of managers are not aware of this program. 17% of the managers stated that there was no precarious work in their context.

Another initiative evaluated in this study was the program for qualification and modernization of HRH decentralized units (nickname PROGESUS - Program of Qualification and Strengthening the Labour and Education Management in the Unified Health System). This is the best known program among the managers (at 77%). It aims to modernize the health departments through training of health professionals on HRH management, development of a national health workforce information system and purchasing equipments.

Although the study shown that 63.2% have an HRH information system (Table 3), the focal groups identified that many health departments had only the payroll and administrative records as information sources for management.

**Table 3:** HRH information system, Brazil, 2008.

HRH information system	capitals	SES	SMS	TOTAL	%
yes	21	21	118	160	63.24

no	2	5	73	80	31.62

don't know	0	0	7	7	2.77

no response	0	1	5	6	2.37

TOTAL	23	27	203	253	100.00

Source: Pesquisa Gestão do Trabalho e da Educação em Saúde. ObservaRH/IMS-UERJ. Brazil, 2008

Although useful, these two sources are centralized in municipal administration, especially in the North, Midwest and Northeast regions of Brazil, limiting the access and use of the data. This problem was aggravated in many places by the lack of local HRH structure.

In this context, HRH information is fragmented, insufficient and depends on rudimentary processes for data collection and analysis, making health workforce planning and recruitment difficult (Table 3).

### Health education management policies

Among the SGTES proposals for linking health and training, it is worth mentioning the training and specialization program of the SUS health workers, undertaken through agreed partnership activities between educational institutions and state and municipal health departments.

These partnerships may be technical, financial or operational and take the form of specializations, introductory or regular/specific training programs, and also internships. These types of partnerships are found in 67.6% of the departments. It was also observed that the main types of partnership consist of specialization courses (61.4%) and internships (56.1%), as shown in Table 4.

**Table 4:** Mechanisms of technical cooperation between SMS/SES and health educational institutions, Brazil, 2008.

Cooperation mechanism	Number	%
Internship	96	56.14

Specialization training course	105	61.40

Regular training course on specific thematic programs	66	38.60

Induction training courses	57	33.33

Source: Pesquisa Gestão do Trabalho e da Educação em Saúde. ObservaRH/IMS-UERJ. Brazil, 2008.

The Program of Permanent Education in health is a professional training program for health workers, which aims to produce changes in professional practices. It was observed that 46% of the health departments participate in this program, promoting training, specialization and postgraduate programs in different areas.

The Health Professional Education Reorientation Program is an initiative aiming to close the gap between health professional education and primary health care needs in Brazil. The program involves three years of financial support for projects, with a potential for transforming the current education model. The processes of reorientation of education in the 'Pró-Saúde' (Pro-Health) program are organized along three axes: theoretical direction, practice scenario and pedagogical direction. Initially, the program included medicine, nursing and dentistry. In the second phase, other healthcare professionals are included. However, the study showed that 60% of HRH managers are not aware of the program.

## Conclusions

The decentralization of the health system in Brazil was established by the Federal Constitution and assured by specific legislation and norms. Considering the size and geographical and political diversity of the country, it is no surprise that the decentralization process did not develop at the same speed everywhere, nor in a uniform manner.

As shown in the study, the area of human resources of the state health departments (SES) and municipal health departments (SMS) of large cities has been, over time, restructuring and developing actions that go beyond the traditional administrative activities. However, after two decades of the SUS having been implemented, there is still a low management capacity in the area of HRH, as demonstrated by low-quality management and the limited use of management tools to support decision making.

This study shows that in HRH management and inter-sectoral relations, the health workers in Brazil make up a contingent of professionals influenced by different systems of policy formulation, with autonomy, direction and particular concerns not governed by sectoral politics. Therefore, any HRH policies should also involve other areas, such as the ministries of education and labour as well as legislative and judiciary bodies.

The results of this evaluation show the evolution of policy previously restricted to the field of 'human resources' (as inputs). It has now expanded to a conceptual model for labour management and health education, identifying progress and setbacks, critical issues and challenges for the consolidation of decentralized model for HRH management.

Overall the results of this analysis show:

• The key role played by the State Health Department (SES) in negotiation and technical cooperation with the municipal health departments (SMS) with respect to the design and development of effective health labour and education management.

• The central role of the federal agency (SGTES) in providing HRH policy incentives through the use of financial, administrative and technical resources.

• The need for the federal agency (SGTES) to monitor and evaluate HRH policies, especially when adapting to different conditions; and when looking for innovations, particularly in health education.

## Competing interests

The authors declare that they have no competing interests.

## Authors' contributions

Both authors participated equally in all stages of preparation of this paper. They read and approved the final manuscript.
